# Filling the agronomic data gap through a minimum data collection approach

**DOI:** 10.1016/j.fcr.2024.109278

**Published:** 2024-03-15

**Authors:** Fatima A.M. Tenorio, Juan I. Rattalino Edreira, Juan Pablo Monzon, Fernando Aramburu-Merlos, Achim Dobermann, Armelle Gruere, Juan Martin Brihet, Sofia Gayo, Shawn Conley, Spyridon Mourtzinis, Nester Mashingaidze, Alex Sananka, Stephen Aston, Jonathan J. Ojeda, Patricio Grassini

**Affiliations:** a Department of Agronomy and Horticulture, University of Nebraska–Lincoln, Lincoln, NE 68583-0915, USA; b International Fertilizer Association, 49 Avenue d′lena, 75116 Paris, France; c Department of Technological Prospective and Research, Buenos Aires Grain Exchange, Buenos Aires, Argentina; d Department of Agronomy, University of Wisconsin-Madison, Madison, WI 53706, USA; e One Acre Fund, Nairobi, Kenya; f Regrow Ag, Brisbane, QLD 4075, Australia

**Keywords:** Data gaps, Agronomic data, Nutrients, Fertilizer, Stratified sampling, Climate zones

## Abstract

**Context:**

Agronomic data such as applied inputs, management practices, and crop yields are needed for assessing productivity, nutrient balances, resource use efficiency, as well as other aspects of environmental and economic performance of cropping systems. In many instances, however, these data are only available at a coarse level of aggregation or simply do not exist.

**Objectives:**

Here we developed an approach that identifies sites for agronomic data collection for a given crop and country, seeking a balance between minimizing data collection efforts and proper representation of the main crop producing areas.

**Methods:**

The developed approach followed a stratified sampling method based on a spatial framework that delineates major climate zones and crop area distribution maps, which guides selection of sampling areas (SA) until half of the national harvested area is covered. We provided proof of concept about the robustness of the approach using three rich databases including data on fertilizer application rates for maize, wheat, and soybean in Argentina, soybean in the USA, and maize in Kenya, which were collected *via* local experts (Argentina) and field surveys (USA and Kenya). For validation purposes, fertilizer rates per crop and nutrient derived at (sub-) national level following our approach were compared against those derived using all data collected from the whole country.

**Results:**

Application of the approach in Argentina, USA, and Kenya resulted in selection of 12, 28, and 10 SAs, respectively. For each SA, three experts or 20 fields were sufficient to give a robust estimate of average fertilizer rates applied by farmers. Average rates at national level derived from our approach compared well with those derived using the whole database ( ± 10 kg N, ± 2 kg P, ± 1 kg S, and ± 5 kg K per ha) requiring less than one third of the observations.

**Conclusions:**

The developed minimum crop data collection approach can fill the agronomic data gaps in a cost-effective way for major crop systems both in large- and small-scale systems.

**Significance:**

The proposed approach is generic enough to be applied to any crop-country combination to guide collection of key agricultural data at national and subnational levels with modest investment especially for countries that do not currently collect data.

## Highlights

• We developed an approach for the collection of agronomic data.
• The approach was validated for fertilizer rates in Argentina, USA, and Kenya.
• A small number of sampling areas were sufficient to estimate fertilizer rates.
• Three experts or 20 fields per sampling area were sufficient for precise estimation.
• The approach can help fill current gaps in agronomic data in a cost-effective way.

## 1 Introduction

Assessing productivity, environmental, and economic performance of cropping systems is constrained by a lack of key agronomic data at adequate spatial resolution and attainable level of disaggregation (*e.g.,* crop yields, nutrient inputs from fertilizer or organic sources, tillage, irrigation, pesticide use, crop residue management, etc.) (Saito et al., 2021). For example, despite the importance of fertilizer data to estimate production costs, nutrient balances and use efficiency, and environmental impact, current global agricultural statistics databases only provide estimates of fertilizer consumption at country level, without distinguishing nutrient inputs among crops (FAOSTAT, http://www.fao.org/faostat/en/#data/ESB). Greater sub-national as well as crop-specific disaggregation is required for research as well as policy applications. The former includes estimates of nutrient budgets, surpluses, and trends in nutrient use efficiency at sub-national to global scales, which, due to the lack of consistent data, are associated with numerous uncertainties (Zhang et al., 2020, Zhang et al., 2021). Likewise, proper targeting of nutrient policies across the whole food chain (Kanter et al., 2020a, Yang et al., 2022), specific regulations and tradeoffs (Kanter et al., 2020b, Mandrini et al., 2022), and sustainable development roadmaps to meet environmental as well as food security goals, require accurate assessment of the status of nutrient use. Unfortunately, only few countries regularly collect such information at the farm level and in a consistent manner. Noticeable examples include the British Survey of Fertiliser Practice, which has been conducted annually since 1992 (https://www.gov.uk/government/collections/fertiliser-usage) or the Agricultural Chemical Use surveys conducted occasionally in the USA (https://www.nass.usda.gov/Surveys/Guide_to_NASS_Surveys/Chemical_Use/index.php). In most countries, however, researchers and policy makers have relied on far less detailed statistical information because farm surveys are expensive or suitable institutions for implementing them on a regular basis do not exist. Often, surveys only cover specific project areas or broader data categories, such as in the Rural Household Multi-Indicator Survey (RHoMIS) (van Wijk et al., 2020). There is also very little usage of non-traditional data sources, such as data that could be obtained from networks of agronomists (crop advisors) or commercial retail, or through crowdsourcing farmers directly.

In the case of data on fertilizer use by crops, the only globally available data source are estimates made for each country, which are obtained through surveys of local experts and published every 3–4 years since 1992 for some countries (Ludemann et al., 2022). The situation is similar for other agronomically, politically, and commercially important crop data. Except for episodic efforts to fill in these data gaps for specific variables and crop-country combinations, we are not aware of any coordinated initiative that has explicitly sought to overcome the lack of key agronomic data at larger scales. Specifically, we are not aware of any explicit effort to develop an approach that can guide efficient collection of relevant agricultural crop management data at a global scale. Thus, developing a cost-effective approach to collect key agronomic data would significantly improve research on many national and global issues, inform investments, and support the development and implementation of better agricultural and environmental policies. To be cost-effective, such an approach should find a balance between achieving an acceptable coverage of the crop producing area while minimizing data collection efforts. Likewise, such approach should seek to derive not only a national average value for a given variable, but also provide estimates at sub-national level as spatial variation in climate and soil within countries typically leads to differences in yield, management practices, and applied inputs. To do so, data collection should be based on a spatial framework that delineates regions based on those factors influencing yield and management practices (*e.g.,* climate, soil). However, current data collection efforts at sub-national level are based on administrative units (*e.g.*, USDA-NASS), which are not helpful at distinguishing regions with different environmental backgrounds (*e.g.,* climate). Finally, an approach to collect agricultural data should be generic and flexible enough so that it can be applied to any crop-country case in the world, incorporate other variables that may influence farmer practices as needed (*e.g.,* water regime, crop sequence, and farmer typology), and allow for regular updates of these data, so that time series can be developed, which, in turn, would help assess trajectories in input use and efficiency and impact of specific policies. Operational feasibility is therefore another important consideration.

Here we present a minimum data collection approach for estimating representative averages of crop inputs and other agronomic attributes for a given country-crop combination. The approach seeks a compromise between deriving robust estimates at national and sub-national levels while reducing data collection efforts. As a proof of concept, we used fertilizer input rates in large-scale (Argentina and USA) and small-scale systems (Kenya) to validate the approach. The goal is to show that a modest investment on data collection efforts can help countries that do not collect data at present to estimate values for key agronomic variables at national and subnational levels. We discuss strengths and limitations of the proposed approach and implications for collection of agricultural data at global scale.

## 2 Materials and methods

### 2.1 Minimum data collection approach development

**Fig. 1:**
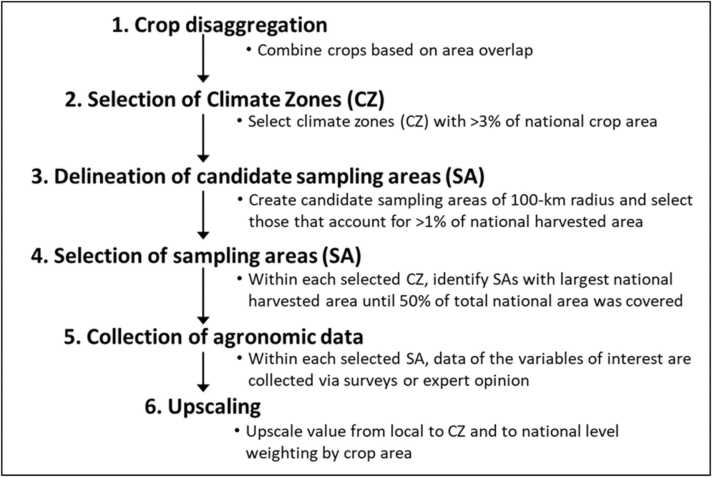
Protocol for a minimum agronomic data collection approach at national level.

**Fig. 2:**
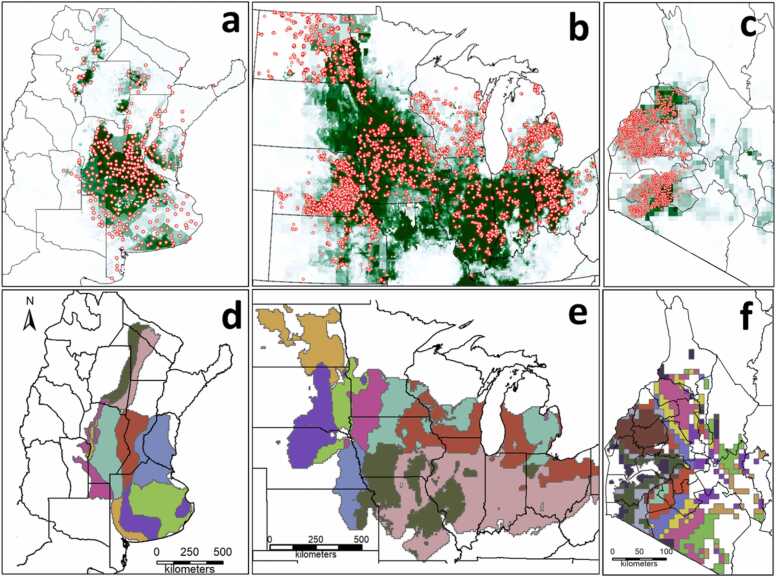
(a, b, c) Location of experts in Argentina (a) and surveyed fields in the USA (b) and Kenya (c) from which data on fertilizer rates were retrieved. The green color shows the combined harvested area for maize, soybean, and wheat in Argentina, soybean in the USA (SPAMv10), and maize in Kenya (SPAMv17, https://www.mapspam.info/data/). (d, e, f) Climate zones in each country, with each color representing a unique climate zone, which, in turn, corresponds to a given combination of growing-degree days, aridity index, and temperature seasonality (Van Wart et al., 2013). Only selected climate zones used in the analysis are shown. Black lines within each country show provinces (Argentina), states (USA), or counties (Kenya) boundaries. Note the different scale for Kenya (c, f).

Our approach builds on the protocol developed by the Global Yield Gap Atlas (GYGA, www.yieldgap.org) for selecting weather stations to estimate yield potential and yield gaps (van Bussel et al., 2015). Our goal was to identify geographic areas, hereafter referred to as ‘sampling areas’ (SA), where agronomic data should be collected for a specific crop in a given country (Fig. 1**,** supplementary Fig. S1). These SAs (*i.e.,* quasi-circle with 100-km radius) are selected based upon (i) the spatial framework delineating climate zones (CZ^3^) developed by GYGA (Van Wart et al., 2013a) and (ii) crop-specific harvested area distribution from SPAM (https://www.mapspam.info/data/). We note that for regions with steep climate gradients (*i.e.*, each CZ that covers a small crop area and cannot accommodate one 100-km radius quasi-circle), a smaller radius (50-km) might be used as we did for Kenya (Fig. 2 **f**).

The approach followed six major steps, and each step has specific rules and thresholds that were defined by iteration, looking for a compromise between reducing data collection efforts while achieving a good representation of the main crop producing areas within the country:

1. **Crop disaggregation:** if the harvested area distribution differs drastically among target crop types, a separate set of SAs should be selected for each crop. If there is substantial overlapping in the harvested area distribution of two or more crop types, then, the same set of SAs can be used to collect data for them. As a generic rule, the same set of SAs are used when overlap in harvested areas between two crops is greater than 66%.
2. **Selection of climate zones (CZ):** the approach identifies CZs that account for > 3% of national crop area (hereafter referred to as ‘selected CZ’). A default value of > 3% reflects the goal of accounting for the area where most of the national production comes from. This value can be adjusted based on crop area coverage target and/or resource availability. For example, if greater coverage is required, this value can be reduced, which would lead to inclusion of CZs that contribute proportionally less to national crop production. In contrast, the value can be increased when less resources are available to collect data, which will lead to greater focus on those CZs that account for the largest share of national crop production.
3. **Delineation of candidate sampling areas:** in each selected CZ, buffers of 100-km radius are created and clipped by the border of the CZs where buffers are located, which helps to minimize climate variation within buffers. As a result, one would expect climate variation within SA to be smaller than variation among SAs. Buffers containing less than 1% of national harvested area are excluded. Remaining buffers (*i.e.,* buffers accounting for at least 1% of national harvested areas) are then referred to as “candidate SAs”. A 100-km radius was used to ensure that there are representative number of fields within a selected SA. We note that selecting buffers smaller than 100-km radius will result in a large number of SAs to achieve the same degree of crop harvested area coverage, ultimately leading to increased data collection efforts. However, in cases where each CZ in a country cannot accommodate one 100-km radius SA, a smaller radius should be used (*e.g.,* 50-km for Kenya).
4. **Selection of sampling areas:** candidate SAs are ranked based on their harvested crop area. The candidate SA with the largest area is selected and overlapping SAs are eliminated. From the remaining SAs, the one with the largest crop harvested area is selected. This process is repeated until selected SAs cover 50% of the total national harvested crop area. At that point, if no SAs have been selected for any of the selected CZs in step (2), the SA with the largest crop harvested area within each of those CZs is selected. If the crop harvested area is less than 50% after SA selection, other candidate SAs located in CZs with < 3% of national crop area are selected until reaching 50% coverage. We note that selecting a variable number of SAs per CZ, instead of a fixed number, ensures that CZs accounting for the largest share of national harvested area are more intensively sampled.
5. **Collection of agricultural data:** Within each selected SA, good quality data on the variables of interest are collected (*e.g., via* surveys or expert opinion). If relevant, these data can be collected separately by water regime (rainfed and irrigated), crop sequences, and, if needed, considering also socio-economic variables (*e.g.,* farmer typology).
6. **Upscaling:** Local estimates within selected SAs are upscaled to sub-national (*i.e.,* CZ) and national level by weighting them based on SA’s harvested area. At the end, the approach provides estimates at CZ and country level for the variable of interest.

Thresholds used for each step of the approach were justified in previous studies and adjusted here as needed. For instance, Van Wart et al. (2013b) and van Bussel et al. (2015) showed that selection of SAs of 100-km radius around weather stations (referred to as ‘buffer’ areas) accounting for > 1% of national harvested area, until covering *ca.* 50% of national harvested area, was sufficient to estimate yield potential and yield gaps at sub-national and national level with a relatively small number of SAs. Hochman et al. (2016) showed that results derived from this approach were remarkably similar to those derived from an intensive data-rich method that used a larger number of SAs. In the case of the threshold used for CZ selection, we reduced the original threshold proposed by van Bussel et al. (2015) from 5% to 3% because the former tends to exclude CZs in less favorable environments for crop production, leading to an overestimation in national-level averages. A smaller threshold (3%) allows selecting CZs that account for largest share of national crop harvested area and obtaining a more accurate averages at national level, without a substantial increase in the number of SAs.

### 2.2 Case studies

We tested the approach using crop nutrient inputs (fertilizer rates) in large scale systems in Argentina and the USA and small-scale system in Kenya as case studies. We used three rich databases with information on: (i) nitrogen (N), phosphorus (P), and sulfur (S) in rainfed wheat, maize, and soybean in Argentina, (ii) P and potassium (K) in rainfed and irrigated soybean in the USA, and (iii) N and P in rainfed maize in Kenya. These country-crop combinations provide an appropriate case study because Argentina includes 5, 6, and 18 million hectares (M ha) sown annually with wheat, maize, and soybean, respectively, while USA includes *ca.* 34 M ha sown with soybean (FAOSTAT, 2021). Likewise, Kenya has *ca.* 2.2 M ha yearly sown with maize. The collected data portrayed well the harvested area in three countries, spreading across many CZs (Fig. 2).

Data from Argentina were collected by ReTAA (or AATS, Applied Agricultural Technology Survey, https://www.bolsadecereales.com/tecnologia-informes) (Fig. 2**a, d**). ReTAA is a major project from the Department of Technological Prospective and Research of the Buenos Aires Grain Exchange (https://www.bolsadecereales.com/), collecting crop management data for the most important crops in Argentina every year. Data are collected *via* phone call interviews with local experts, who are agronomists, ag retailers, and extension educators spread across the country. Each expert reports average yield, inputs, and management practices for their ‘area of influence’, which means that data from one expert is an average from several fields. In the case of fertilizers, data on \N, P, and S fertilizer application were collected for each crop. Data were disaggregated by early and late seasons in the case of soybean and maize. No data on K fertilizer rate were collected for any crop as this nutrient is rarely applied to maize, soybean, and wheat in Argentina and the same occurred in the case of N in soybean. All data were collected from rainfed crops as the irrigated area sown with maize, soybean, and wheat in Argentina is negligible. Details about the ReTAA approach to collect data is provided elsewhere (https://www.bolsadecereales.com/tecnologia-informes). For this study, we used data provided by *ca.* 300 experts annually during three crop seasons (2016/2017 - 2018/2019).

In the case of the USA, soybean producers provided field-level data *via* returned surveys distributed by local crop consultants, extension educators, soybean grower boards, and Natural Resources Districts (Fig. 2**b, e**). Each soybean producer reported yield, applied fertilizer rates, and other management practices for fields sown with soybean in each year. Details on survey and database are available elsewhere (Rattalino Edreira et al., 2017, Rattalino Edreira et al., 2020, Mourtzinis et al., 2018; https://coolbean.info/wp-content/uploads/sites/3/2019/02/2019_Soybean_Benchmarking_ReviewFinal.pdf). On average, the database contained *ca.* 2000 fields per year over four crop years (2014–2017). For our analysis, we used data on P and K fertilizer rates as these are the two most common nutrients applied in soybean in the USA.

Data from Kenya was collected by One Acre Fund (One Acre Fund, 2021), which is a non-governmental organization that provides smallholder farmers access to agricultural training, credit, crop insurance services, and farming supplies, which allows them to improve their technology adoption. One Acre Fund collected data on yield and management practices like fertilizer inputs, fertilization method, planting dates, seed type, incidence of pest and diseases, and field size. Input rates were calculated as the ratio of the self-reported total input amount and field size. Here, we used data on N and P fertilizer rates annually reported by *ca.* 770 farmers across five crop seasons (2016–2020) (Fig. 2**c, f**). We note that while One Acre Fund data included both farmers who subscribed to the “One Acre Fund Program” and non-subscribed farmers from neighboring farms, for our analysis, we only included the latter group to avoid biases in inputs use. We focused on western Kenya, which represents 60% of maize area in the country, since One Acre Fund database was concentrated in this region.

Fertilizer rates are reported in all cases as kilograms of elemental nutrient per hectare per crop. We made our best effort to evaluate the quality of our databases by comparing the average national fertilizer rates against independent estimates in Argentina (Fertilizar; https://fertilizar.org.ar/), in the USA (USDA-NASS; https://www.nass.usda.gov/), and in Kenya (FAOSTAT, https://www.fao.org/faostat/en/#data/ESB, https://www.fertilizer.org/) for each crop by nutrient combination (Table 1). However, a one-to-one match of the data was impossible given differences in years and aggregation. In Argentina, average (2016/2017 - 2018/2019) national fertilizer rates from the database were compared against estimates from Fertilizar, which were only available for the 2014/2015 crop season. In the case of USA, we used the averages from the most recent (2015 and 2017) USDA-NASS data on fertilizer rates (https://www.ers.usda.gov/data-products/fertilizer-use-and-price/) for the comparison. For Kenya, averages of national fertilizer rates between 2016 and 2020 were compared against estimates from FAOSTAT for the same year, however FAOSTAT only reported applied fertilizer rates per cropland.

**Table 1:** Comparison for average fertilizer rates at national level between the database used in our studies *versus* independent estimates reported by others (Fertilizar in Argentina, USDA-NASS in USA, and FAOSTAT in Kenya) for each country-crop-nutrient combination. Database estimates are averages over 2017–2019 (Argentina, n = 1855), 2014–2017 (USA, n = 8015) and 2016–2020 (Kenya, n = 3849) while the independent estimates correspond to values in year 2014 (Argentina), 2-y (2015 & 2017) averages (USA), and 5-y (2016–2020) averages (Kenya). Parenthetic values are the standard error of the mean, which are only shown for the database values as were not reported for the other estimates.

Country	Crop	Nutrient^†^	Database (kg ha^−1^)	Other estimates (kg ha^−1^)
Argentina	Wheat	N	55 ( ± 0.9)	63
		P	12 ( ± 0.2)	16
	Maize	N	58 ( ± 0.8)	44
		P	12 ( ± 0.2)	11
	Soybean	P	5 ( ± 0.1)	5
USA	Soybean	P	8 ( ± 0.3)	10
		K	29 ( ± 0.8)	32
Kenya	Maize^‡^	N	25 ( ± 0.4)	24
		P	16 ( ± 0.2)	10

^†^ N: nitrogen; P: phosphorous; K: potassium (all expressed in kg of elemental nutrient).

^‡^ FAOSTAT reported nutrients applied (kg ha^−1^) per cropland in Kenya.

### 2.3 Application and evaluation of the minimum data collection approach

The approach described in Section 2.1 was applied to select SAs in Argentina, USA and Kenya. For the selection, we considered the harvested crop area around year 2010 for Argentina and USA and year 2017 for Kenya (https://www.mapspam.info/data/). Rainfed wheat, maize, and soybean harvested areas in Argentina largely overlap (67%). Hence, we used the combined harvested area for the three crops in Argentina. In the case of soybean in the USA, we used the combined rainfed and irrigated area, with the latter accounting for *ca.* 10% of the national soybean area. Average fertilizer rates were estimated for each SA in each year using all available experts (Argentina) or fields (USA and Kenya) within each SA. In the case of Argentina, fertilizer rates reported for early- and late-sown maize and soybean were weighted by their respective area within each SA to calculate an average rate for each crop. Likewise, for those SAs in the USA where both irrigated and rainfed soybean production exist, an average fertilizer rate was estimated by weighting for the harvested area under each water regime. Because fertilizer application rates can fluctuate over time due to changes in grain and fertilizer prices, our comparisons are based on the average fertilizer rate per SA calculated based on three (Argentina), four (USA), or five (Kenya) crop seasons.

We evaluated our approach for data collection by estimating average fertilizer rates at national and subnational levels. To do so, we compared estimates of fertilizer rates at CZ and country level as derived from the selected SAs *versus* those estimated using all available observations from the three databases for all country-crop-nutrient combinations (Fig. 5**, S3**). For the latter, we first weighted each numerator (Argentina) or field (USA) by the relative share of national crop area as determined from the crop area within a 25-km buffer (large-scale) or 1-km (small-scale) centered at each expert or field and then calculated the average value for each CZ. Subsequently, to upscale from CZ to national level, we averaged CZ estimates, weighting the CZ estimates by their respective crop harvested area, separately for each crop. In the case of the USA and Kenya, only CZs with at least 10 fields per CZ per year were considered for the evaluation of our approach. We used root mean square error (RMSE), relative RMSE (rRMSE, *i.e*., RMSE as a percentage of the national average using all available observations), and mean absolute error (MAE) to evaluate the degree of agreement between fertilizer rates estimated *via* our approach *versus* those estimated using all available observations in the database. Finally, to evaluate capacity of the approach to collect other agronomically relevant data besides nutrient fertilizer inputs, we replicated our analysis using yield data available in the databases from the three countries, following the same methodology described for nutrient inputs (Fig. S4).

### 2.4 Data requirement per sampling area

A key question is the minimum number of experts or fields per SA that is needed to retrieve a robust estimate of fertilizer rate at national level. To answer this question, we ran our data collection approach using different sample sizes per SA (*n*) (Fig. 6**, S5**). In the case of Argentina, we varied the number of experts per SA from one to 16. For the USA and Kenya, sample sizes ranged from one to 50 fields per SA. Because different subsets of experts or fields in the same SA can lead to different estimates, we repeated the analysis using independent samples for a given sampling size. To do this, we performed bootstrap analysis, which is a resampling technique used to estimate statistics on a population by sampling a dataset (Simpson and Mayer-Hasselwander, 1985). A bootstrap sample is a random sample selected with replacement from the original statistical observations, which means that some of the original observations can be repeated more than once or omitted from an individual bootstrap sample (Dixon, 2002). For each size sample size, 200 bootstrap samples were used to compute different national fertilizer rate averages for a given crop and nutrient. These 200 averages derived *via* bootstrapping were used to compute 95% confidence intervals (CI) by removing the 2.5% lowest and highest values. The margin of error was then calculated as half of the CI width, which represents the uncertainty of national level fertilizer estimates due to variation in fertilizer rates across experts (Argentina) or fields (USA and Kenya) within the same SA. For comparison purposes, we considered that a given sample size was appropriate when the margin of error of national-level estimates were within ± 10 kg N ha^−1^, ± 2 kg P ha^−1^, ± 5 kg K ha^−1^, and ± 1 kg S ha^−1^. We determined these thresholds based on what we believed are agronomically relevant differences. For example, 10 kg N ha^−1^ is roughly equivalent to half a ton of cereal grain. Further, once the minimum number of experts (Argentina) or fields (USA and Kenya) per SA required for precise estimation of fertilizer rate was established, we compared the averages obtained with our minimum data collection approach against the national fertilizer rate retrieved from the whole database and the number of surveyed field or experts required to reach those numbers with a reasonable level of precision (Table 2). This analysis was performed separately for each crop-nutrient combination. Finally, to assess the degree of precision at sub-national level, we repeated the analysis for the climate zones accounting for largest maize, wheat, and soybean area in Argentina, for largest soybean area in USA, and for largest maize area in Kenya **(**Fig. S6**)**.

**Table 2:** National average fertilizer rates estimated using all experts (Argentina) or fields (USA and Kenya) available in the databases and confidence intervals (CI) for those estimated following our minimum data collection approach based on three experts or 20 fields in each of the 12, 28, and 10 selected sampling areas in Argentina, USA, and Kenya, respectively. Estimates are averages over 2017–2019 (Argentina), 2014–2017 (USA), and 2016–2020 (Kenya). The number (n) of local experts (Argentina) or fields (USA and Kenya) used to compute each average or CI is shown.

Country	Crop	Nutrient	Whole database			Minimum data collection approach	
			n	Average (kg ha^−1^)		n	CI (kg ha^−1^)^†^
Argentina	Wheat	N	1855	55		36	52 – 65.1
		P		12			11.3 – 14.6
		S		1			0.6 – 2.3
	Maize	N		58			47.4 – 63.9
		P		12			9.7 – 15.1
		S		2			0.8 – 3.1
	Soybean	P		5			3.5 – 6.4
		S		2			1.5 – 3.6
USA	Soybean	P	8015	8		560	7 – 10.3
		K		29			23.3 – 34
Kenya	Maize	N	3849	25		200	20 – 29.5
		P		16			13 – 17.6

†95% confidence interval (CI) for a set of 100 estimates of national average fertilizer rates based on random samples of three experts or 20 fields per sampling area. Rates are reported in kg elemental nutrient: nitrogen (N), phosphorous (P), potassium (K), and sulfur (S).

## 3 Results

### 3.1 Database quality assessment

Comparison of average fertilizer rates between our database and other available sources showed reasonable agreement between databases at national level in Argentina and USA (Table 1). Across country-crop-nutrient combinations in the two large-scale system countries, differences between databases were within ± 14 kg N, 4 kg P, and 3 kg K per ha, indicating that the databases used for our study portrayed well the variation in fertilizer rates across country-crop-nutrient combinations. In the case of smallholder fields in Kenya, our database had comparable N rate and a slightly higher (+6 kg ha^−1^) P relative to FAOSTAT data. The discrepancy can be attributed to FAOSTAT reporting nutrient rates per ha of cropland and to the location of our study area (western Kenya) which has favorable conditions for crop condition, thus, nutrient rates are expected to be higher.

### 3.2 Application of the minimum data collection approach in Argentina, USA, and Kenya

**Fig. 3:**
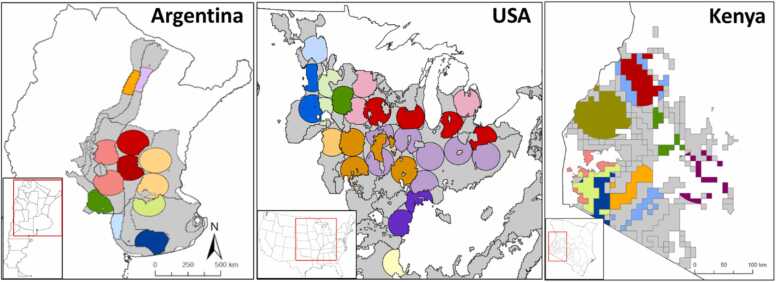
Selected sampling areas (SAs) following the minimum data collection approach for wheat, maize, and soybean in Argentina (left), soybean in USA (middle), and maize in Kenya (right). Quasi-circles represent selected SAs and those with same color belong to the same climate zone. Inset shows the location of the study region within each country. Note the different scale for Kenya.

Our approach selected 9 CZs in Argentina,11 CZs in the USA, and 9 CZs in Kenya, resulting in 12, 28, and 10 SAs, respectively (Fig. 3). The selected CZs represented 91% of the aggregated wheat, maize, and soybean harvested areas in Argentina, 76% of the soybean harvested area in the USA, and 74% of maize harvested area in western Kenya. While selected SAs represented *ca.* 50% of the combined national harvested area for the target crops in each country. Hence, our approach achieved a large coverage of the crop harvested area with a relatively small number of SAs, for countries that included 29 M ha (Argentina), 34 M ha (USA), and 2.2 M ha (Kenya) sown with the target crops. Selected SAs in Argentina included 45% of the total number of experts in the database while 51% and 63% of the total number of fields available in USA and Kenya databases, respectively. In most cases, selected SAs had a radius smaller than 100 km, as originally delineated for SA selection, because they were clipped by the borders of the CZs. On average, there were 25 experts (Argentina), 40 fields (USA), and 300 fields (Kenya) per SA.

### 3.3 Validation of the approach at CZ and national level

**Fig. 4:**
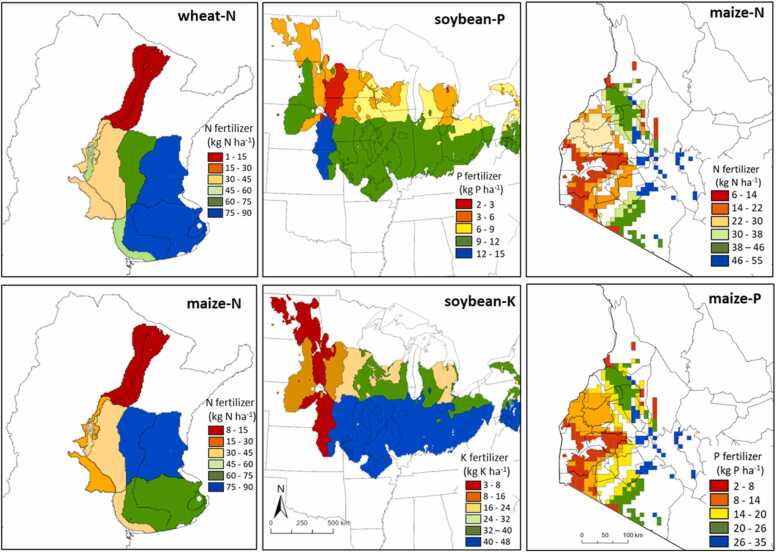
Examples of average fertilizer rate per climate zone estimated based on the minimum data collection approach for nitrogen (N) in wheat and maize in Argentina (left panels), and phosphorous (P) and potassium (K) for soybean in USA (middle panels), and N and P for maize in Kenya (right panels).

**Fig. 5:**
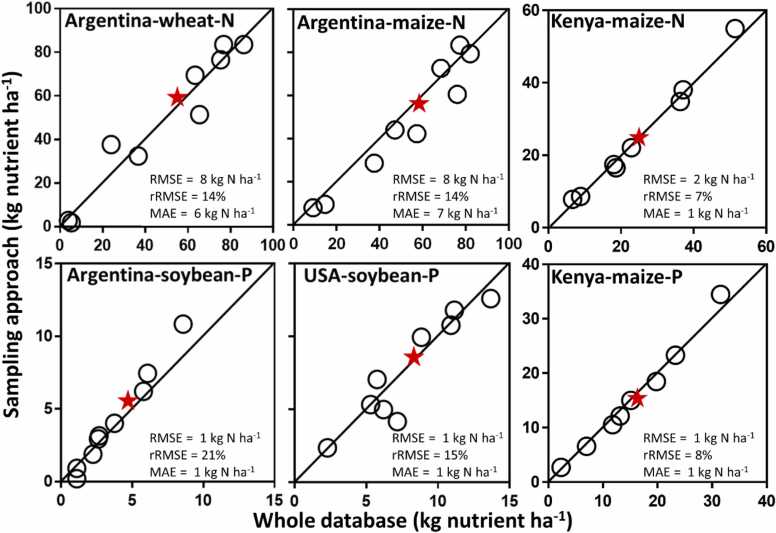
Comparison of fertilizer rates retrieved from our minimum data collection approach and those estimated using all available data for nitrogen (N) in wheat and maize in Argentina, and maize in Kenya, while phosphorous (P) for soybean in Argentina and USA, and maize in Kenya. Circles represent average fertilizer rates for each selected climate zone while the red stars show national averages. In all cases, values are averages over three (Argentina), four (USA), or five crop seasons (Kenya). Root mean square error (RMSE), relative RMSE (rRMSE), mean absolute error (MAE), and the y = x line is shown.

Our minimum data collection approach derived estimates of fertilizer rates that were comparable to those based on the entire database, both at sub-national (*i.e.* CZ) and national levels for N in wheat and maize in Argentina, P in soybean in Argentina and USA, and for N and P in maize in Kenya (Fig. 4, Fig. 5**, S2).** For example, overall RMSE for N fertilizer rates across selected CZs for wheat in Argentina was 8 kg N ha^−1^, representing 14% of the national N fertilizer rate based on all experts. In the case of P fertilizer rate for soybean in the USA, the RMSE was 1 kg P ha^−1^, representing 15% of the national average based on all surveyed fields. Similarly for N in Kenya, RMSE represents only 8% of the national N fertilizer across all fields. A strong agreement at national and CZ level was also found for other country-crop-nutrient combinations, with RMSE representing 15% or less of national fertilizer rates based on whole database, except for S in Argentina due to lower rates (supplementary Fig. S3). Across all country-crop-nutrient combinations, there was no indication of consistent differences in RMSE, expressed as percentage of the overall averages, among crops, nutrients, or regions. Besides the strong agreement, our approach was able to reproduce well the variation in fertilizer rate across CZs, for example, from near zero up to 80 kg N ha^−1^ for maize in Argentina. For all country-crop-nutrient combinations, the slope of the fitted linear regression did not deviate from one (*p* > 0.17), indicating no bias in the estimation of fertilizer rates across the selected CZs. Our approach also reproduced averages and variation in grain yield, with average RMSE representing 9% of national yield level based on the whole database (Supplementary Fig. S4). This was expected since nutrient inputs are typically correlated with yield. Overall, our analysis suggests that the approach can be used to collect a wider range of agronomically relevant variables.

In relation to national averages, fertilizer rates estimated based on our approach were always ± 4 kg nutrient ha^−1^ ( ± 10%) of those estimated using the entire databases. Except for N in maize in Argentina and P in maize in Kenya, national estimates based on experts or fields within selected SAs were slightly higher (range: 0.2–4 kg nutrient ha^−1^) than those based on all experts or fields included in the databases. Our method tended to sample more intensively areas with greater crop harvested area and yield, which also tended to have higher fertilizer rates. Nevertheless, our approach resulted in similar estimates of fertilizer rates by using only, on average, half of the experts or fields included in the databases.

### 3.4 Influence of number of experts or fields per sampling area

**Fig. 6:**
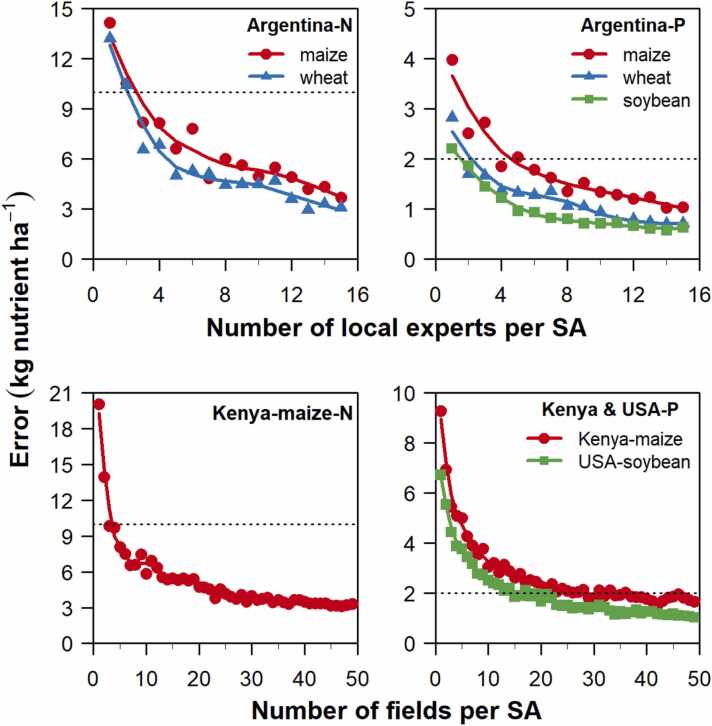
Margin of error of national averages of fertilizer rates estimated with different number of experts (1 to 16, Argentina) or fields (1 to 50, USA and Kenya) per selected sampling area (SA) for nitrogen (N) for wheat and maize in Argentina, phosphorous (P) for wheat, maize, and soybean in Argentina, N and P for maize in Kenya, and P soybean in USA. The margin of error of fertilizer rates for a given sample size (n) was estimated based on 200 randomly selected subsets of experts or fields of size n. Also shown are dashed lines indicating ± 10 kg N ha^−1^ and ± 2 kg P ha^−1^deviations, which are considered here to be reasonable levels of precision.

In Argentina, three experts per SA were sufficient to deliver national fertilizer rates with a margin of error lower than (or close to) 10 kg N ha^−1^ and 2 kg P ha^−1^, which are considered here to be reasonable levels of precision for agronomic applications (Fig. 6). In the case of soybean in the USA and maize in Kenya, we found that 20 fields per SA were adequate to derive average national fertilizer rates within ± 10 kg N ha^−1^ and ± 2 kg P ha^−1^ of estimates based on all available fields in the selected SAs. The same result was also found for other crop-nutrient combinations, with national fertilizer rate estimates within ± 2 kg P ha^−1^, ± 1 kg S ha^−1^, and ± 5 kg K ha^−1^ (supplementary Fig. S5). The number of experts and fields per SA required for robust estimation of fertilizer rates as estimated here (3 and 20, respectively) was considerably smaller than the average number of experts in Argentina and fields per SA in USA and Kenya included in the databases (25, 40, 300 respectively). An assessment performed at CZ level for a subset of crop-country combination revealed that reaching the desired precision would be difficult, even when using large sample sizes, meaning that there will be higher uncertainty at sub-national level **(**supplementary Fig. S6**)**.

Combining the results on selected SAs (12, 28, and 10 in Argentina, USA, and Kenya respectively) and required number of experts (3) or fields (20), one can estimate that a total of 36 experts (Argentina), 560 fields (USA), and 200 fields (Kenya) would be needed to estimate average fertilizer application rates at national and subnational levels with a reasonable level of precision (Table 2). The required number of experts and fields represented *ca.* 12%, 28%, and 26% of those included in the entire databases for Argentina, USA, and Kenya, respectively. Average national fertilizer rates estimated following our approach (*i.e.,* using 36 experts in Argentina, 560 fields in USA, and 200 fields in Kenya) were comparable to those derived using the entire databases, across all country-crop-nutrient combinations. In most cases, CIs of fertilizer rates estimated by our approach were within (or close to) ± 10 kg N ha^−1^, ± 2 kg P ha^−1^, ± 1 kg S ha^−1^, and ± 5 kg K ha^−1^ of the average values derived from the whole databases.

## 4 Discussion

While new techniques such as remote sensing, crowdsourcing, block chain, and data mining may one day allow collection of agricultural data at little cost, our study shows that a modest investment on ‘smart’, geospatially-based data collection based on a stratified sampling strategy, as routinely done in other disciplines (Goodbody et al., 2023), would allow retrieving average values for key agronomic variables at national and sub-national level within a relatively short timeframe. For example, in large producing countries like Argentina and the USA, orienting data collection to 12 and 28 sampling areas, respectively, strategically selected based on agroclimatic conditions and crop harvest area distribution, delivered robust estimates of fertilizer rates at national levels **(**Fig. 3, Fig. 5**)**. Furthermore, we showed that a total of 36 experts (Argentina) or 560 fields (USA) were sufficient for estimating national average nutrient fertilizer rates with a reasonable level of precision **(**Table 2**)**. These values are remarkable considering that each of these countries includes *ca.* 30 M ha sown with the target crops. Similarly, application of the approach in small scale systems like Kenya showed that data collection across 20 fields for each of the 10 SAs (*i.e.,* 200 fields annually) showed comparable fertilizer rates to those estimated from 770 surveyed fields per year. Overall, our study showed that it is possible to generate robust estimates of fertilizer rates with a relatively small number of experts or field surveys, which is particularly relevant for countries that have limited resources for data collection.

We note that extending our validation approach to other parts of the world where current data availability is scarce may require modifications in the proposed methodology. For example, the spatial stratification method used here could be modified further by including other criteria that may enable better representation of more diverse soils, cropping systems, and farms. In addition, while fertilizer and yield were used here for proof of concept, the approach can be used for collection of many other types of agronomic data, particularly crop inputs and agronomic practices that are needed to capture heterogeneity within CZ or SA as driven by other biophysical and/or socio-economic factors. Whereas specific thresholds for number of experts or survey can vary among variables, region, and specific goal, we do not see any reason why the overall framework cannot be used for collection of other agronomically relevant data such as pesticide use, straw management, plant densities, *etc.*, as variation in these variables are typically related to the same biophysical factors that influence variation in yield and fertilizer inputs. In our study, we used fixed thresholds to determine the required sample size based on what we believe agronomically relevant differences are (*i.e*., ± 10 kg N, ± 2 kg P, ± 1 kg S, and ± 5 kg K per ha). Ultimately, the sample size can be modified depending upon the user-desired level of precision following the relationships shown Fig. 6. Likewise, increasing sample size and/or number of SAs per CZ may be desirable when the goal is to derive sub-national estimates with a precision comparable to that set as target in the present study (*i.e.*, ± 10 kg N, ± 2 kg P, ± 1 kg S, and ± 5 kg K per ha) **(**supplementary Fig. S6**)**. Beyond these potential adjustments, our approach serves a first step to fulfill the current agronomic data gap for major crop systems around the world using a transparent, objective, and systematic approach that orients data collection to specific geographic areas in each country and, by doing so, minimizing the required resources needed to support these efforts.

Average values at country level typically mask large spatial variation in agricultural practices within countries. For example, the national average N rate for wheat in Argentina was *ca.* 60 kg N ha^−1^, varying from *ca.* 2 to 80 kg N ha^−1^ across CZs (Fig. 4, Fig. 5). Similar patterns were found for all country-crop-nutrient combinations **(**supplementary Fig. S3**)**. Our approach overcomes the limitation of current global databases by providing estimates for the most important crop producing regions within a country and capturing the most influential environmental gradients that drive differences in management and yield. Such spatial granularity is useful for informing research and extension programs at sub-national level. For example, if data on nutrient inputs is complemented with yield data, one could identify areas where nutrient balances are excessive or deficient, pointing out opportunities to reduce the negative environmental impact or avoid soil degradation (Riccetto et al., 2020). To summarize, having access to agricultural data for major CZs within a country can help to better target investments on agricultural research and development programs and orient policy, which would not be possible when only aggregated data at national level are available.

The proposed method is flexible to further disaggregate data collection in each SA based on other criteria (*e.g.,* farmer typology, soil type, water regime, cropping system). In Argentina, for example, local experts collect data separately for different cropping systems such as late *versus* early maize and soybean. Our results indicated that average N fertilizer rates were higher in early-sown maize (60 kg N ha^−1^) than in late-sown maize (54 kg N ha^−1^). In the case of rainfed and irrigated soybean in USA, we did not find differences in national average P and K fertilizer between water regimes. Likewise, the proposed method can accommodate other socio-economic variables that influence farmers’ technologies and decision making. For example, in oil palm farming in Indonesia, average yield and applied inputs are different between smallholders and large plantations, regardless of the biophysical background (Monzon et al., 2021). In those cases, it would be relevant to collect data separately for the two farmer typologies, which both may occur in the same CZ. It may also be desirable to bring other biophysical variables into our spatial framework if evidence exists that management practices and applied inputs would be influenced by them and associated spatial data are available. For example, soil properties could be incorporated into the spatial framework to account for possible changes in applied inputs and yield among soil types (*e.g.*, Rezaei et al., 2015; Rattalino Edreira et al., 2018; Ojeda et al., 2021). We note, however, that addition of other variables needs to be carefully evaluated considering the expected added value and the extra data collection efforts involved. The capability of our approach to further disaggregate data collection based on both biophysical and socio-economic background provides enough flexibility to be applicable globally, but we also argue to keep it as simple and robust as possible. As a next step, we aim to expand our work to more countries, crops and agronomically important variables, giving priority to crop-regions combinations accounting for largest fraction of global crop production.

## 5 Conclusions

We developed a minimum crop data collection approach that seeks a balance between robust estimation of key agronomic attributes and reduced efforts in data collection both in large- and small-scale systems. The approach delivers national and sub-national estimates of agronomic variables using a reasonable number of SAs per country and a relatively small number of experts or fields per SA. In our case studies for fertilizer rates, the approach selected 12 (Argentina), 28 (USA), and 10 SAs (Kenya) that represented 50% of national production for a given crop. Only three experts or 20 fields per SA seemed sufficient for robust estimation of average national rates. The approach is generic and flexible enough so that it can be applied in other crop-countries and data can be further disaggregated by water regime, crop cycle, and socio-economic parameters.

## CRediT authorship contribution statement

**Sananka Alex:** Data curation, Writing – review & editing. **Tenorio Fatima:** Conceptualization, Formal analysis, Investigation, Writing – original draft, Writing – review & editing. **Mashingaidze Nester:** Data curation, Writing – review & editing. **Ojeda Jonathan J.:** Data curation, Writing – review & editing. **Monzon Juan Pablo:** Data curation, Investigation, Methodology, Writing – review & editing. **Aston Stephen:** Data curation, Writing – review & editing. **Rattalino Edreira Juan I.:** Conceptualization, Data curation, Formal analysis, Investigation. **Aramburu-Merlos Fernando:** Formal analysis, Writing – review & editing. **Grassini Patricio:** Conceptualization, Data curation, Formal analysis, Funding acquisition, Investigation, Methodology, Project administration, Supervision, Writing – original draft, Writing – review & editing. **Gruere Armelle:** Data curation, Writing – review & editing. **Dobermann Achim:** Conceptualization, Methodology, Writing – review & editing. **Gayo Sofia:** Data curation, Investigation, Writing – review & editing. **Brihet Juan Martin:** Data curation, Investigation, Writing – review & editing. **Mourtzinis Spyridon:** Data curation, Writing – review & editing. **Conley Shawn:** Data curation, Writing – review & editing.

## Declaration of Competing Interest

The paper content has not been previously published nor is under consideration for publication elsewhere. All co-authors have contributed to the paper and have agreed to be listed as coauthors.

## Data Availability

Data will be made available on request.

## Footnotes

3 A CZ corresponds to a unique combination of growing degree days (which determines the length of the growing season), aridity index (which is a proxy to water availability), and temperature seasonality (which distinguishes regions with different seasonal variation in temperature). Generally, a CZ refers to a geographic area with homogeneous climate conditions.

## References

[bib1] Dixon, P.M., 2002. Bootstrap resampling. Encyclopedia of Environmetrics 1, 212– 220.

[bib2] FAOSTAT, 2021. Crops and Livestock Trade Database. https://www.fao.org/faostat/en/#home. Accessed on July 26, 2021.

[bib3] Fertilizar. Fertilizar Associacion Civil. https://fertilizar.org.ar/. Accessed on January 4, 2022.

[bib4] Goodbody T.R.H., Coops N.C., Queinnec M., White J.C., Tompalski P., Hudak A.T., Auty D., Valbuena R., LeBoeuf A., Sinclair I., McCartney G., Prieur J.-F., Woods M.E. (2023). sgsR: a structurally guided sampling toolbox for LiDAR-based forest inventories. Forestry.

[bib5] GYGA. Global Yield Gap Atlas. https://www.yieldgap.org/. Accessed on June 28, 2020.

[bib6] Hochman Z., Gobbett D., Horan H., Navarro Garcia J. (2016). Data rich yield gap analysis of wheat in Australia. Field Crops Res.

[bib7] Kanter D.R., Bartolini F., Kugelberg S., Leip A., Oeneme O., Uwizeye A. (2020). Nitrogen pollution policy beyond the farm. Nat. Food.

[bib8] Kanter D.R., Chodos O., Nordland O., Rutigliano M., Winiwarter W. (2020). Gaps and opportunities in nitrogen pollution policies around the world. Nat. Sustain..

[bib9] Ludemann C.I., Gruere A., Heffer P., Dobermann A. (2022). Global data on fertilizer use by crop and by country. Sci. Data.

[bib10] Mandrini G., Pittelkow C.M., Archontoulis S., Kanter D., Martin N.F. (2022). Exploring trade-offs between profit, yield, and the environmental footprint of potential nitrogen fertilizer regulations in the US midwest. Front. Plant Sci..

[bib11] Monzon J.P., Slingerland M.A., Rahutomo S., Agus F., Oberthür T., Andrade J.F., Couëdel A., Edreira J.I.R., Hekman W., Beuken R.V.D., Hidayat F., Pradiko I., Purwantomo D.K.G., Donough C.R., Sugianto H., Lim Y.L., Farrell T., Grassini P. (2021). Fostering a climate-smart intensification for oil palm. Nat. Sustain..

[bib12] Mourtzinis S., Rattalino Edreira J.I., Grassini P., Roth A.C., Casteel S.N., Ciampitti I.A., Kandel H.J., Kyveryga P.M., Licht M.A., Lindsey L.E., Mueller D.S., Nafziger E.D., Naeve S.L., Stanley J., Staton M.J., Conley S.P. (2018). Sifting and winnowing: analysis of farmer field data for soybean in the US north-central region. Field Crops Res.

[bib13] Ojeda J.J., Rezaei E.E., Kamali B., McPhee J., Meinke H., Siebert S., Webb M.A., Ara I., Mulcahy F., Ewert F. (2021). Impact of crop management and environment on the spatio-temporal variance of potato yield at regional scale. Field Crops Res.

[bib14] One Acre Fund (2021). Data from MEL agronomic surveys in Kenya, Rwanda, Uganda, Burundi. Malawi Tanzan..

[bib15] Rattalino Edreira J.I., Mourtzinis S., Conley S.P., Roth A.C., Ciampitti I.A., Licht M.A., Kandel H., Kyverga P.M., Lindsey L.E., Mueller D.S., Naeve S.L., Nafziger E., Specht J.E., Stanley J., Staton M.J., Grassini P. (2017). Assessing causes of yield gaps in agricultural areas with diversity in climate and soils. Agric. . Meteorol..

[bib16] Rattalino Edreira J.I., Cassman K.G., Hochman Z., Van Ittersum M.K., Van Bussel L., Claessens L., Grassini P. (2018). Beyond the plot: technology extrapolation domains for scaling out agronomic science. Environ. Res. Lett..

[bib17] Rattalino Edreira J.I., Mourtzinis S., Azzari G., Andrade J.F., Conley S.P., Specht J.E., Grassini P. (2020). Combining field-level data and remote sensing to understand impact of management practices on producer yields. Field Crops Res.

[bib18] ReTAA A.A.T.S., Applied Agricultural Technology Survey. Buenos Aires Grain Exchange. https://www.bolsadecereales.com/tecnologia-informes. Accessed on June 28, 2020.

[bib19] Rezaei E.E., Siebert S., Ewert F. (2015). Impact of data resolution on heat and drought stress simulated for winter wheat in Germany. Eur. J. Agron..

[bib20] Riccetto S., Davis A.S., Guan K., Pittelkow C.M. (2020). Integrated assessment of crop production and resource use efficiency indicators for the U.S. Corn Belt. Glob. Food Secur..

[bib21] Saito K., Six J., Komatsu S., Snapp S., Rosenstock T., Arouna A., Cole S., Taulya G., Vanlauwe B. (2021). Agronomic gain: definition, approach, and application. Field Crops Res..

[bib22] Simpson G., Mayer-Hasselwander H. (1985). Bootstrap sampling: applications in gamma-ray astronomy. Astron. Astrophys..

[bib23] USDA-NASS. USDA National Agricultural Statistics Service. https://www.nass.usda.gov/. Accessed on November 17, 2021.

[bib24] van Bussel L.G.J., Grassini P., Van Wart J., Wolf J., Claessens L., Yang H., Boogaard H., de Groot H., Saito K., Cassman K.G., van Ittersum M.K. (2015). From field to atlas: Upscaling of location-specific yield gap estimates. Field Crops Res.

[bib25] Van Wart J., van Bussel L.G.J., Wolf J., Licker R., Grassini P., Nelson A., Boogaard H., Gerber J., Mueller N.D., Claessens L., van Ittersum M.K., Cassman K.G. (2013). Use of agro-climatic zones to upscale simulated crop yield potential. Field Crops Res.

[bib26] Van Wart J., Kersebaum K.C., Peng S., Milner M., Cassman K.G. (2013). Estimating crop yield potential at regional to national scales. Field Crops Res.

[bib27] van Wijk M., Hammond J., Gorman L., Adams S., Ayantunde A., Baines D., Yameogo V. (2020). The Rural Household Multiple Indicator Survey, data from 13,310 farm households in 21 countries. Sci. Data.

[bib28] Yang A.L., Raghuram N., Adhya T.K., Porter S.D., Panda A.N., Kaushik H., Jayaweera A., Nissanka S.P., Anik A.R., Shifa S., Sharna S.C. (2022). Policies to combat nitrogen pollution in South Asia: gaps and opportunities. Environ. Res. Lett..

[bib29] Zhang X., Davidson E., Zou T., Lassaletta L., Quan Z., Li T., Zhang W. (2020). Quantifying nutrient budgets for sustainable nutrient management. Glob. Biogeochem. Cycles.

[bib30] Zhang X., Zou T., Lassaletta L., Mueller N.D., Tubiello F.N., Lisk M.D., Davidson E.A. (2021). Quantification of global and national nitrogen budgets for crop production. Nat. Food.

