# Supplementary material for: Filling the agronomic data gap through a minimum data collection approach
Source: Field Crops Res. 2024 Mar 15;308:109278. doi: 10.1016/j.fcr.2024.109278 (PMC10933791; doi:10.1016/j.fcr.2024.109278)
Supplement: Supplementary file 1 — Supplementary material [file mmc1.docx]

**SUPPLEMENTARY MATERIALS**

*
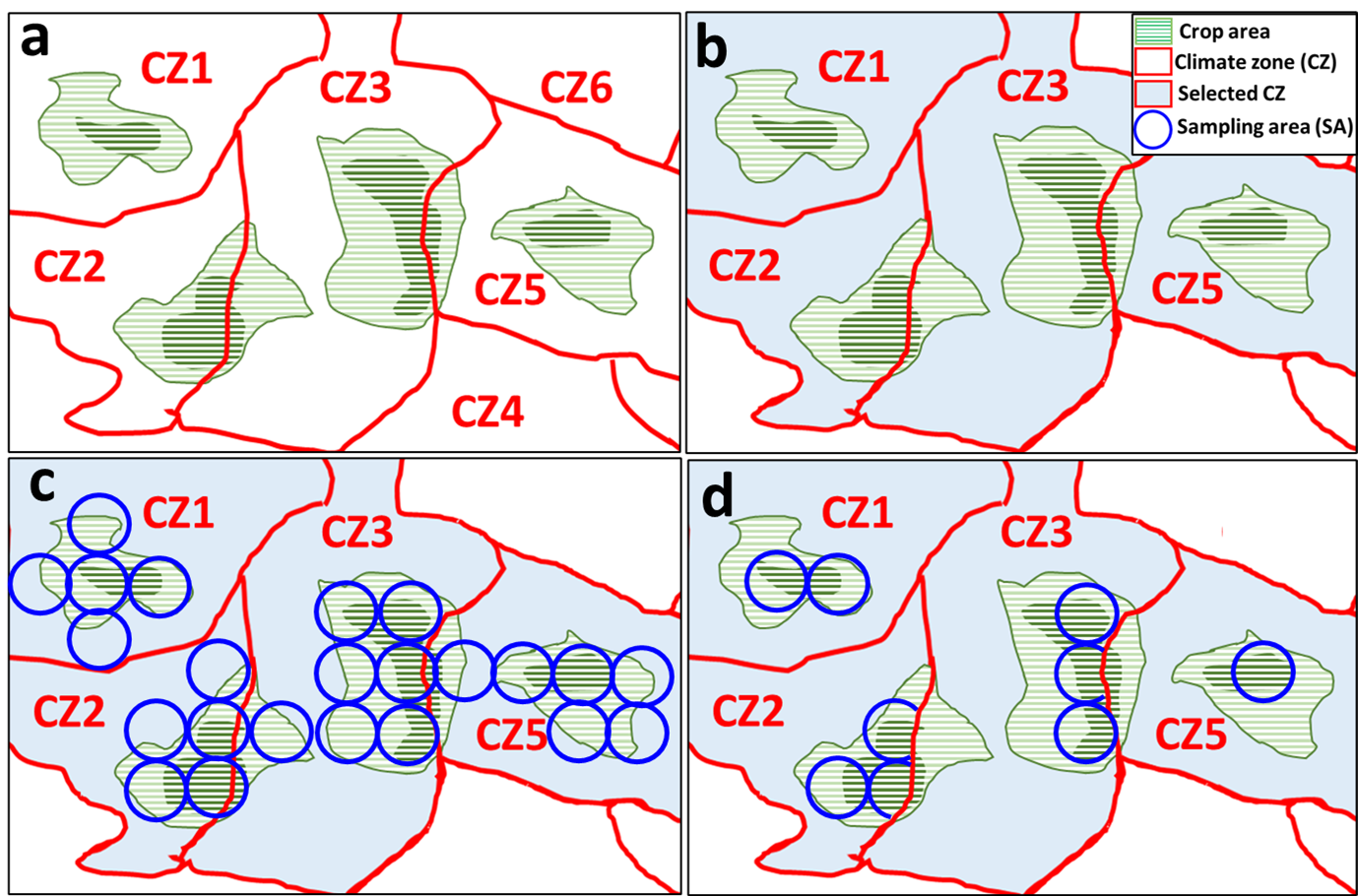
*

***Figure S1****. Schematic illustration of the protocol to select sampling area for a hypothetical crop-country combination, including (a) climate zones (CZ) and crop area (greener area means more intensive crop harvested area), (b) selection of CZs that account for >3% of national crop harvested area, (c) candidate sampling areas (SAs) accounting for >1% of national harvested area in each of the selected climate zones; and (d) selection of the final SAs where to orient data collection.*


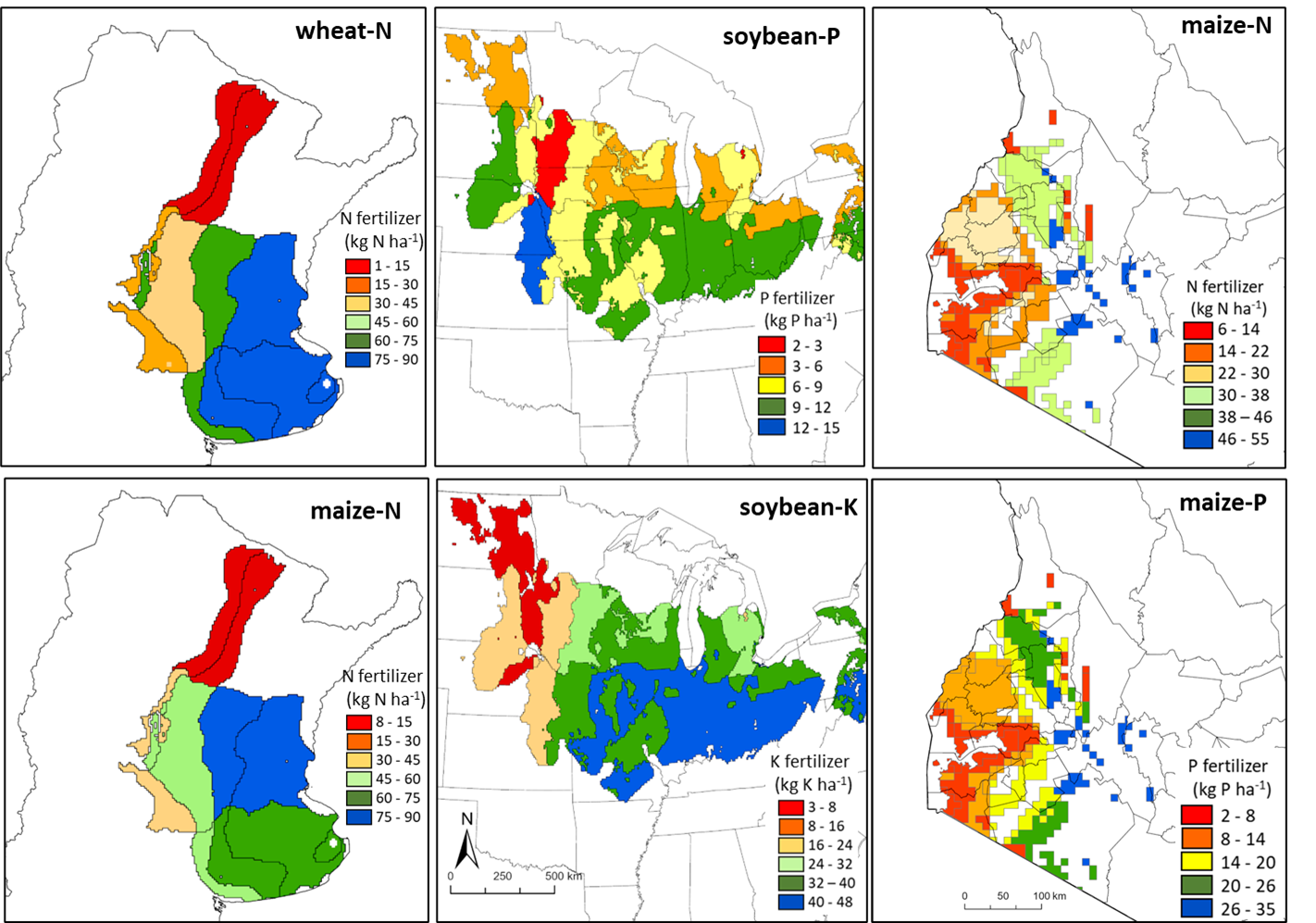


***Figure S2.*** *Examples of average fertilizer rate per climate zone estimated based on all enumerators or fields in the database for nitrogen (N) in wheat and maize in Argentina (left panels), and phosphorous (P) and potassium (K) for soybean in USA (middle panels), and N and P for maize in Kenya (right panels).*


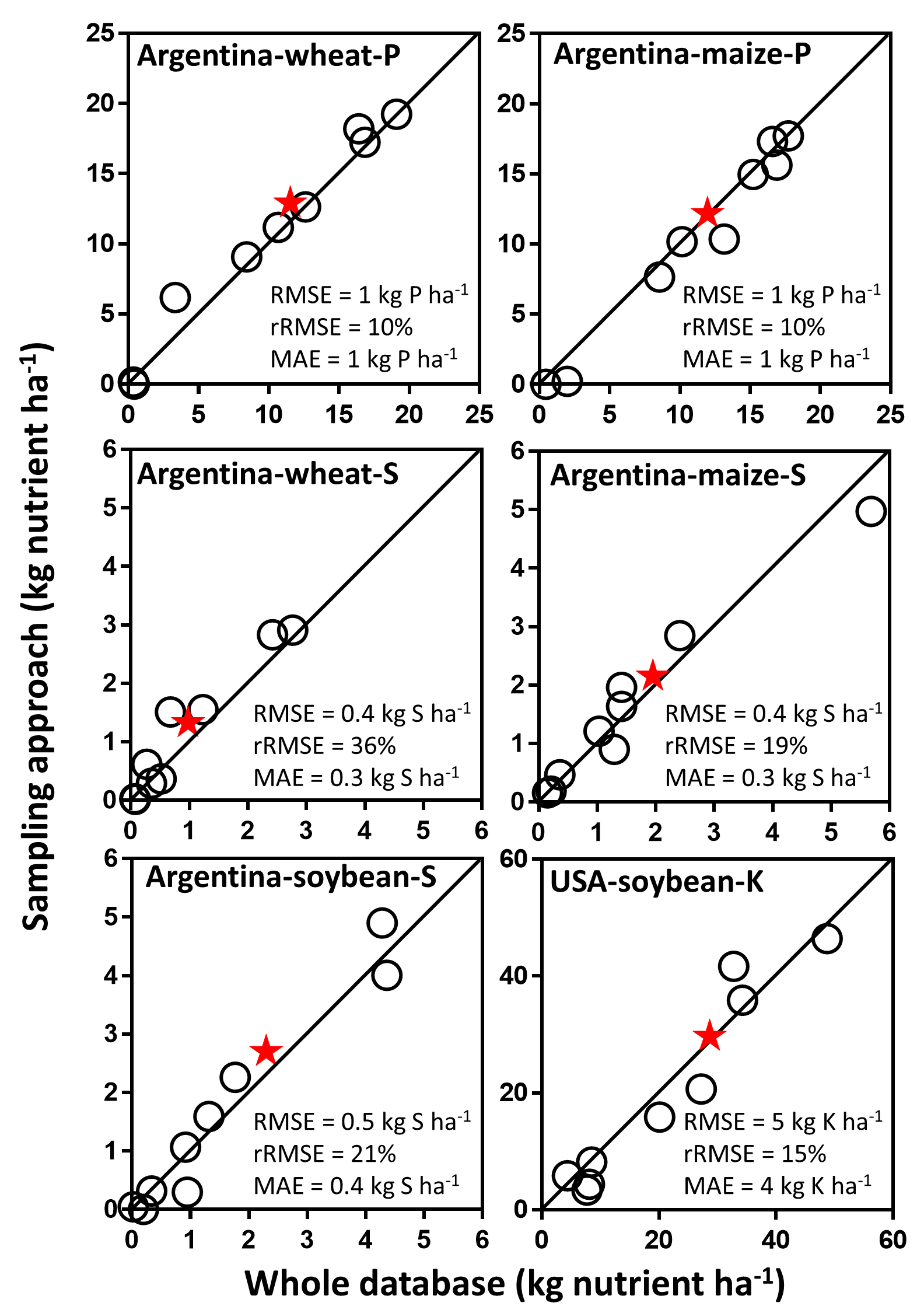


***Figure S3.*** *Comparison of fertilizer rates retrieved from our minimum data collection approach and those estimated using all available data for phosphorous (P) and sulfur (S) in wheat, maize, and soybean in Argentina and potassium (K) for soybean in the USA. Circles represent average fertilizer rates for each selected climate zone while the red stars show national averages. In all cases, values are averages over three (Argentina) or four crop seasons (USA). Root mean square error (RMSE), relative RMSE (rRMSE), mean absolute error (MAE), and the y=x line is shown.*


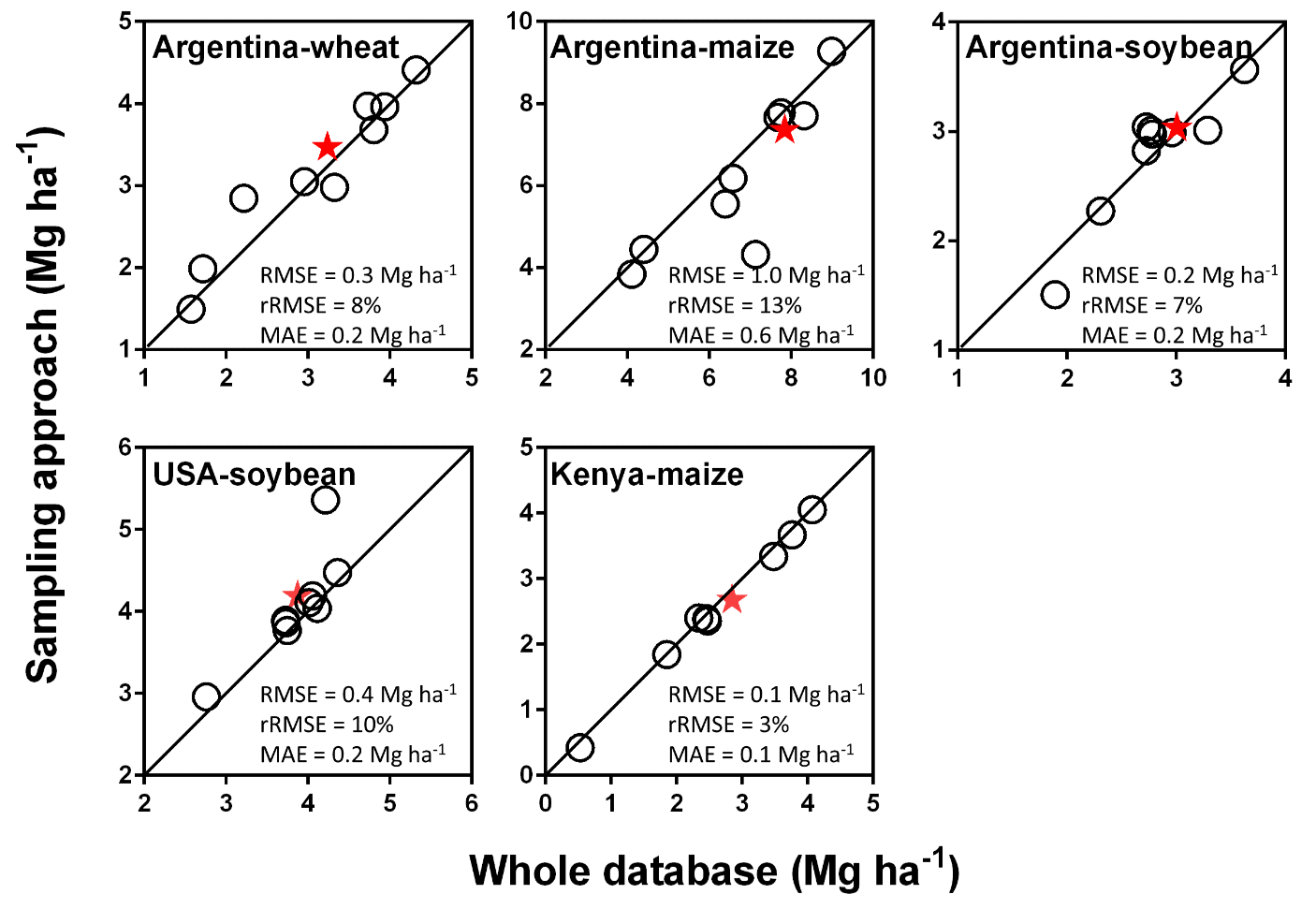


***Figure S4.*** *Comparison of average crop yields retrieved from our minimum data collection approach and those estimated using all available data for wheat, maize, and soybean in Argentina, soybean in USA, and maize in Kenya. Circles represent average crop yields for each selected climate zone while the red stars show national averages. In all cases, values are averages over three (Argentina), four (USA), or five crop seasons (Kenya). Root mean square error (RMSE), relative RMSE (rRMSE), mean absolute error (MAE), and the y=x line are shown.*

***
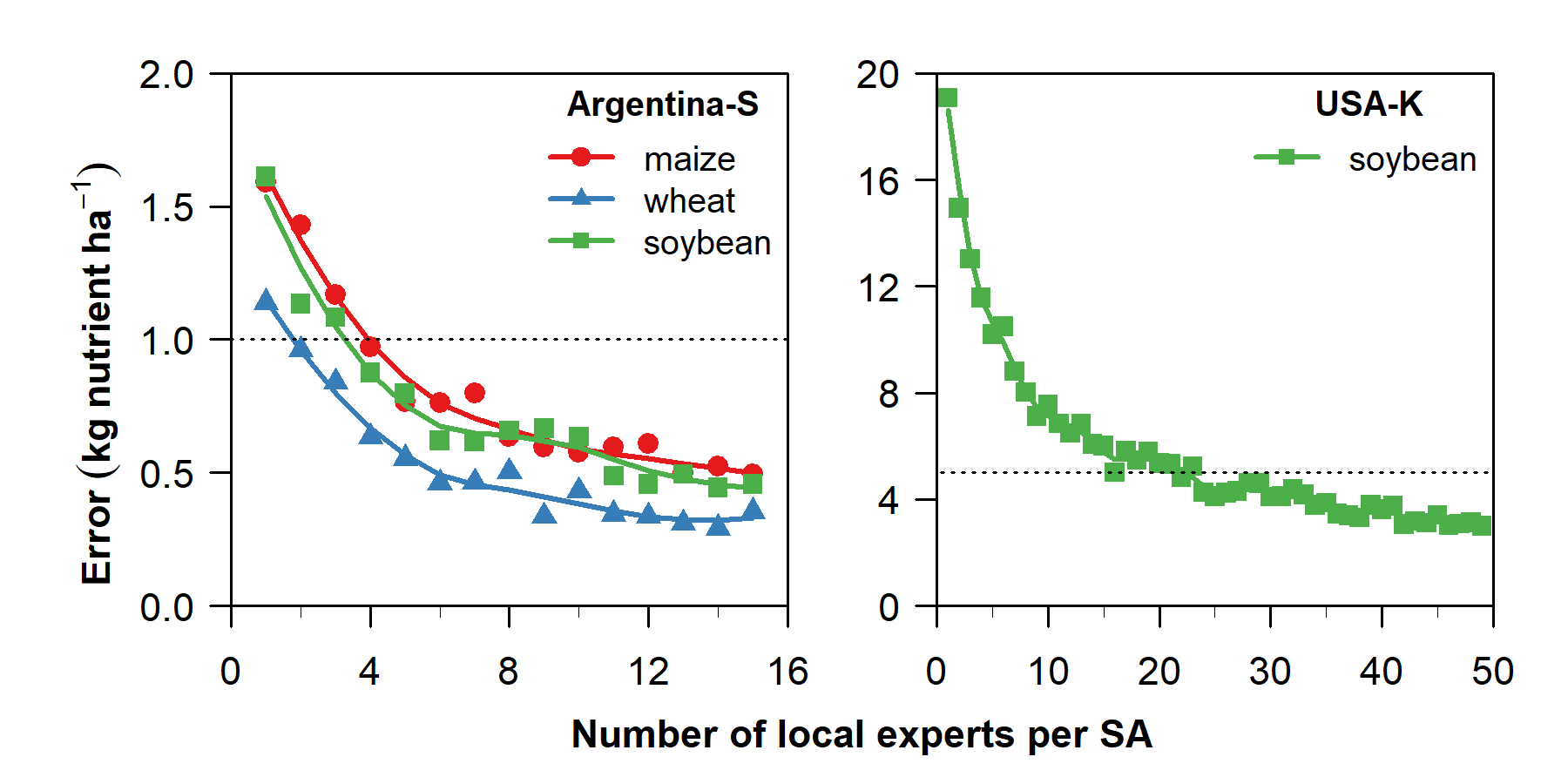
***

***Figure S5.*** *Margin of error of national averages of fertilizer rates estimated with different number of enumerators (1 to 16, Argentina) or fields (1 to 50, USA) per selected sampling area (SA) for sulfur (S) in wheat, maize, and soybean in Argentina and potassium (K) for soybean in the USA. The margin of error of fertilizer rates for a given sample size (n) was estimated based on 200 randomly selected subsets of enumerators or fields of size n. Also shown are dashed lines indicating ±1 kg S ha^-1^ and ±5 kg K ha^-1^deviations, which are considered here to be reasonable levels of precision.*


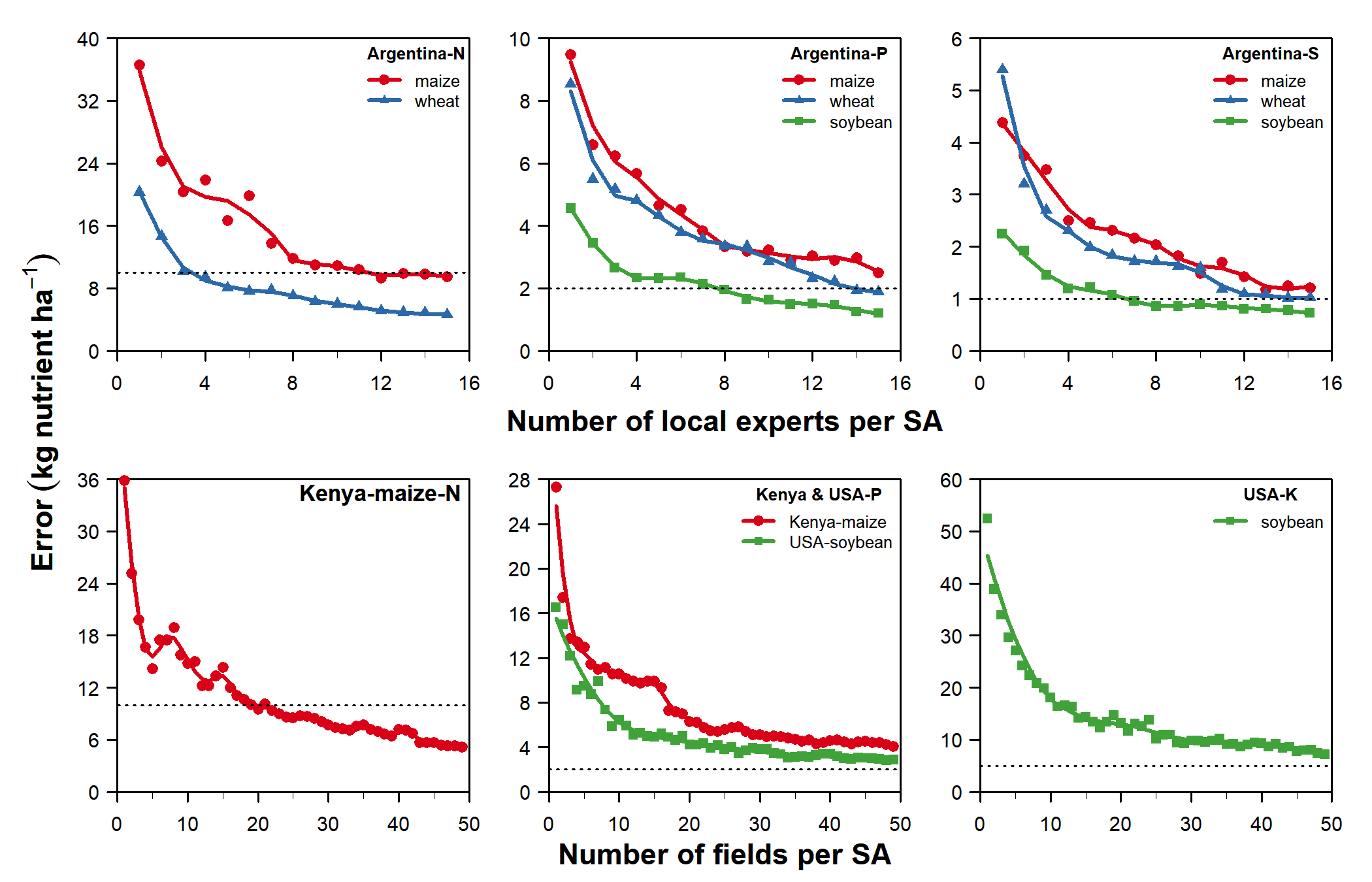


***Figure S6.*** *Margin of error of national averages of fertilizer rates estimated with different number of enumerators (1 to 16, Argentina) or fields (1 to 50, USA and Kenya) per selected sampling area (SA) for nitrogen (N), phosphorous (P) and sulfur (S) for the main climate zones where wheat, maize, and soybean are grown in Argentina, P and potassium (K) for the main climate zone where soybean is grown in the USA, and N and P for the main climate zone where maize is grown in Kenya. The margin of error of fertilizer rates for a given sample size (n) was estimated based on 200 randomly selected subsets of enumerators or fields of size n. Also shown are dashed lines indicating ±10 kg N ha^-1^, ±2 kg P ha^-1^, ±5 kg K ha^-1^, and ±1 kg S ha^-1^ deviations, which are considered here to be reasonable levels of precision.*
